# The politics of human embryo research and the motivation to achieve PGD

**DOI:** 10.1016/j.rbmo.2011.01.008

**Published:** 2011-05

**Authors:** Anastasia A. Theodosiou, Martin H. Johnson

**Affiliations:** Anatomy School and Trophoblast Research Centre, Department of Physiology, Development and Neuroscience, Downing Street, Cambridge CB2 3DY, UK

**Keywords:** Enoch Powell, HFE Act, history, human embryo research, PGD

## Abstract

This article reports a historical study of factors influencing the achievement of clinical preimplantation genetic diagnosis (PGD) in 1990, 22 years after its first demonstration in animals. During the 1970s, research on PGD continued in large farm animals, but serious interest in human PGD was not evident until 1986. First, interest in PGD during the 1970s waned with the advent of prenatal testing, which for gynaecologists was clinically more familiar, technically simpler and ethically less challenging than IVF. Indeed, IVF was viewed with widespread suspicion until the first IVF births in 1978. Second, interest in clinical PGD was stimulated by the UK Parliamentary reaction against human embryo research that greeted the Warnock Report in 1984. This hostility led scientists to initiate a pro-research campaign, further galvanized in 1985 by MP Enoch Powell’s bid to ban such research. However, while Powell abhorred embryo research, he approved of PGD, a stance that divided the anti-research lobby. Accordingly, the campaigners for research emphasized that it was needed to achieve PGD. Powell demanded evidence of such projects and PGD research increased from 1986. It is concluded that UK political debates on embryo research played a critical role in stimulating the achievement of clinical PGD.

Human pregnancies following preimplantation genetic diagnosis (PGD) for embryo sex were announced in 1990, 22 years after the technique was pioneered in animals. PGD in humans required not only technological advances, such as IVF and sensitive diagnostic tests, but also the motivation to develop and apply them. Our historical analysis shows that, although research on PGD continued in large farm animals during the 1970s, and techniques of the required sensitivity were developed on mouse embryo models, interest in clinical PGD was not evident until 1986. Two factors stimulated this sudden change in motivation. First, interest in PGD was depressed during the 1970s by the advent of prenatal diagnostic techniques, which for gynaecologists were clinically, technically and ethically less challenging than IVF. IVF was then regarded with a suspicion that only started to wane in the early 1980s following the first IVF births. Second, the UK Parliamentary reaction against human embryo research that greeted the Warnock Report in 1984 provided a positive stimulus to clinical PGD by prompting scientists to form a pro-research lobby, which was further galvanized in early 1985 by MP Enoch Powell’s almost-successful bid to ban human embryo research. We show that while Powell abhorred embryo research, he approved of PGD, a stance that fractured the unity of the anti-research lobby. Accordingly, the pro-research lobby emphasized that embryo research was needed to achieve PGD. Powell demanded evidence of such projects, thereby, we argue, stimulating PGD research from 1986. Our evidence shows that UK political debates about PGD played a critical role in stimulating the achievement of PGD clinically.

## Introduction

In 1990, the UK Parliament voted in favour of a Bill to allow the generation and use of human embryos *in vitro*, including their regulated use in research and assisted reproduction (HFE Act, 1990). Mulkay (1997), in his influential account of the late 1980s debates leading to the passage of this Act, attributes a key role to preimplantation genetic diagnosis (PGD) in shaping the form of this legislation (pp. 132–133). He claims that supporters of embryo research convinced MPs that ‘many forms of genetic disorder could be more or less eradicated by means of genetic screening of IVF embryos’ (p. 41). Indeed, just 5 days before the 1990 Commons vote, a team from the Hammersmith Hospital led by Robert Winston announced the first clinical pregnancy following use of PGD to avoid transmission of a sex-linked genetic disease (Handyside et al., 1990). Winston himself suggested ‘parliamentary opinion had been decisively altered… by this clear demonstration that research on human embryos really does produce genuine therapeutic benefits’ (cited in Mulkay, p. 42). Likewise, Franklin and Roberts (2006, p. 39), in their ethnography of PGD in the UK, confirm the ‘decisive role’ of the technique-in-the-making that was ‘hailed as “winning the vote”’ by ‘focusing and clarifying public attitudes’. They describe the powerful ‘bridging capacity’ of PGD between ‘the promise of almost immediate future benefit’ and the need for ‘considerable ongoing scientific research’ at the time (p. 58).

This paper traces the early history of PGD from demonstration in principle in animal studies in 1968 to clinical application in 1990, and asks the question: what influenced its development as a medical technology? Research shows that not only did innovations in medical technology shape Parliamentary decisions, but that the political debate itself exerted a critical motivational stimulus to achieve PGD clinically.

## Materials and methods

### Sources

The Hansard database, containing verbatim records of UK Parliamentary debates, was used to explore the role of PGD in the embryo research debate from the discussions of the Warnock Report (Warnock, 1984) to those of the HFE Bill in 1990. These debates are cited by speaker and year in the text (e.g. Lord Ennals: Hansard, 1990), and by a complete online database reference in the reference list.

Archival material was accessed from the UK Medical Research Council (MRC) records at the National Archives at Kew, Surrey, UK (NA); from the papers of Professor Peter Braude and the Progress Educational Trust papers held at the London School of Economics’ Archive (LSE); from the Anne McLaren papers at the British Library (BL); and from Enoch Powell’s personal papers in the Churchill College Archives, Cambridge (CC). These archival sources comprise folders or boxes, each containing up to several hundred unnumbered items. These sources are cited with a numbered archival code in the order that they appear in the text (NA1–5; LSE1–14; BL1–3 and CC1–10) and are included in a separate archival reference list as a short description, date and archival location by box/folder division and subdivision, where applicable.

This study also draws on information from unpublished interviews by Professors Martin Johnson and Sarah Franklin with: Dame Mary Warnock, Chair of the Committee of Enquiry into Human Fertilisation and Embryology from 1982–1984; Mrs Jenny C Croft, the secretary to the Committee; and Roy Cunningham, Assistant Secretary at the Department of Health.

### Historical nomenclature

Preimplantation genetic diagnosis (PGD) now refers to the clinical technique of diagnosing genetic characteristics in preimplantation embryos without precluding viability or further embryonic and fetal development following transfer to a receptive uterus. Before being applied clinically in 1990, several different names were used to describe the concept underlying this technique. For example, initial animal studies referred mainly to ‘embryo sexing’ (Hare and Betteridge, 1978). The terms ‘preimplantation diagnosis’ (McLaren, 1985), ‘preimplantation genetic diagnosis’ (Penketh and McLaren, 1987) and ‘embryo biopsy’ (CC1) all appeared in the literature between 1985 and 1990, along with ‘prenatal diagnosis before implantation’ (McLaren, 1985) and ‘preimplantation genetic screening’ (Whittingham and Penketh, 1987). It is unclear when the acronyms PID, PGD and PIGD were first introduced, although a PubMed search did not reveal their published use before 1993 (PID; Holding et al., 1993), 1994 (PGD; Verlinsky and Kuliev, 1994) and 1997 (PIGD; Davis, 1997). However, as PubMed searches return only hits from the title and abstract (where available) and do not include other sources such as newspaper articles, it is possible that these acronyms were used earlier. Harper (2009, p. 5) suggests that the concept was referred to as ‘preimplantation diagnosis’ or ‘PID’ in many of the first papers, but that the name ‘was changed’ to PGD ‘by people entering the field later on to avoid confusing the acronym PID with that for pelvic inflammatory disease’.

### Criteria for the PGD concept

Since the underlying concept of PGD went by several different names before 1990 and appeared in a variety of contexts, a source was considered to be pertinent to PGD if it demonstrated one or both of two criteria: (i) a desire or intent to diagnose preimplantation embryos without precluding further development or transfer, even if the methods or results were not compatible with these aims (e.g. studies on gametes or on whole embryos but with reference to the possibility of embryo biopsy); and (ii) methods or results describing diagnosis without precluding further development or transfer, even if the desire or intent for embryo transfer was absent (e.g. sexing embryo biopsies to study X-inactivation and gene dosage, rather than for commercial or clinical diagnostic incentives).

## Results

### Technological development

The earliest reference to the concept of PGD dates to 1965 and to Robert Edwards (1965), who was to achieve IVF in humans in 1969 and led to the birth of Louise Brown in 1978 (Johnson et al., 2010). In 1967, the concept of PGD was fleshed out experimentally in an attempt to sex in-vivo generated rabbit blastocysts, by using euchrysine vital staining and fluorescence microscopy to visualize sex chromatin (a nuclear inclusion of the condensed and inactive X chromosome, indicating female sex; Edwards and Gardner (1967)). However, this technique, involving the exposure of the whole embryo to fluorescent light, was potentially mutagenic and not safely compatible with embryo transfer. The following year, Gardner and Edwards (1968) micro-surgically biopsied 200–300 trophoblast cells, stained and examined them and then transferred the ‘sexed’ biopsied blastocysts to pseudopregnant recipients. The sex of the developed fetuses was confirmed anatomically and histologically at full term, providing ‘a reliable method of sexing’ which was ‘compatible with further development’. They also addressed the potential for clinical application, stating that the ‘scope of experimental embryology could be greatly extended’ to include the detection of ‘autosomally inherited deformities from either parent’ (Anon, 1968; Edwards and Gardner, 1968; Gardner and Edwards, 1968). Twenty-two years later, this pioneering study was successfully translated to the clinic.

A conventional understanding is that technological problems had to be solved for this translation to happen. Thus, the human embryo is smaller and has fewer cells than the rabbit embryo and its sex chromatin cannot be visualized as easily, necessitating the use of more sensitive, reliable and discriminating diagnostic technologies (Table 1). Indeed, Harper (2009, p. 4) suggests that the ‘challenge of the introduction of molecular biology for PGD was the move from working with millions of cells to the very few cells of the embryo’. As late as 1985, it was ‘generally agreed that there were no single-cell diagnostic techniques available, and that the biopsied cell(s) would have to be cultured to obtain sufficient cells for the diagnosis’ (Harper, 2009, p. 3). In addition, it was considered ‘unlikely’ that diagnosis and transfer could be achieved within the same menstrual cycle and that clinical PGD would thus ‘depend on the successful application of embryo cryopreservation’ (West et al., 1987). Successful freezing with subsequent thawing, transfer and clinical pregnancy was described for animals by Whittingham et al. (1972) and for humans by Trounson and Mohr (1983). In fact, however, the first clinical application of PGD used fresh embryos and employed the polymerase chain reaction (PCR) to amplify DNA specific to the Y chromosome (Handyside et al., 1990).

A second technical obstacle was the need for enough human embryos to develop PGD technology. Animal embryos – and with difficulty human embryos (Table 2) – could be obtained by lavage of the uterus and oviducts *in situ* or by flushing removed organs. IVF provided an alternative source of embryos, although this was not demonstrated convincingly in humans until 1969 (Edwards et al., 1969) and the first IVF births not achieved until 1978 (Edwards and Steptoe, 1978). Moreover, although human blastocysts were produced after IVF as early as 1971 (Steptoe et al., 1971), their routine production was considered too difficult, meaning that only cleavage-stage biopsy could be contemplated thereby providing even fewer cells for testing. Even by 1969, the generation of animal fetuses and live young by IVF and embryo transfer was not routine, having been achieved only in rabbits (Chang, 1959), hamsters (Yanagimachi and Chang, 1963) and mice (Whittingham, 1968). Thus, it has been argued that species differences between human and rabbit embryos necessitated technological developments and that the lack of research embryos and appropriate diagnostic techniques impeded successful research on the clinical application of PGD in humans for 22 years.

### The technological explanation evaluated

Undoubtedly technological limitations were problematic. However, motivation was required to overcome these problems. What evidence of such motivation exists and when is it first apparent?

#### Experience with preimplantation sexing in animals

In 1978, the control of the sex ratio in cattle and other farm animals was described as an alluring ‘old dream’ (cited in Hare and Betteridge, 1978), due to the potential commercial advantages of selectively producing dairy cattle and tender beef and preventing the occurrence in multiple pregnancies of free martins (female fetuses partially masculinized by exposure to adjacent male fetuses; Winterberger-Torres and Popescu, 1980). Pre-fertilization sperm sexing had been described as the ‘ideal method of controlling the sex ratio’ in cattle and was pursued through the 1970s and 1980s by investigating whether X- and Y-bearing spermatozoa might be discriminated on the basis of mass, volume, charge or motility, but these approaches were largely unsuccessful (Van Vliet et al., 1989). Indeed, sexing of preimplantation embryos was singled out as seeming ‘more encouraging’ agriculturally (Hare and Betteridge, 1978) and a number of experimental studies were reported in the 1970s and early 1980s.

Following the 1968 rabbit study, the next reports on the PGD principle were in sheep (cited in Polge and Rowson, 1975) and cattle (Hare et al., 1976), both of which produce large preimplantation embryos with many trophoblast cells. Trophoblast biopsies were sexed from metaphase spreads and the diagnosis confirmed by embryo transfer. Hare et al. (1976) reported confident sexing of 20/34 biopsied embryos (58.8%) but could not sex 11 due to absent or poor metaphase spreads. Sex was confirmed correctly following birth, slaughter or spontaneous abortion in 7/8 cases. Two later studies also used metaphase spreads on fewer cells biopsied from earlier embryos. Moustafa et al. (1978) biopsied only 7–10 blastomeres per bovine embryo, and determined sex successfully in 63% of 29 day-6 morulae and 53% of 15 day-7 early blastocysts. The authors reported ‘no deleterious effect on the subsequent embryonic and fetal developments’ following biopsy and transfer to a recipient uterus. In 1983, Severova and Dyban claimed successful sexing in mice by isolating and culturing a single blastomere at the 4-cell stage, the remaining three cells developing into a normal blastocyst. Both these later studies suggested an application in farm animals, and, crucially, both demonstrated approaches to PGD that required neither PCR nor hundreds of cells, unlike the methods of Handyside et al. (1990) and of Gardner and Edwards (1968), respectively. However, none of these four accounts referred to the possibility of pursuing PGD clinically in humans, nor of its use to avoid genetic disease. In contrast, Modlinski and McLaren (1980), using polar body karyotyping in an attempt to assess the status of the maternal chromosomal complement in the embryo, did refer to the possibility of clinical PGD, but were pessimistic about its likely usefulness.

Embryo typing by enzyme micro-assay was described by Epstein et al. (1978), who separated mouse blastomeres at the 2-cell stage and cultured each to give ‘twin half-embryos’. One of these halves was sexed by assaying for the X-linked enzyme hypoxanthine-guanine phosphoribosyltransferase (HPRT) and the other by karyotyping. Although this sensitive enzyme assay, used in combination with ‘embryo biopsy by splitting’, demonstrated the feasibility of PGD in the absence of PCR, the authors made no reference to the principle of PGD or to transferring an untested ‘half-embryo’, but were focused on understanding the processes of X inactivation and gene dosage in preimplantation embryos. Around the same time, Monk and her colleagues increased the sensitivity of her enzyme micro-assays sufficient to type single cells, but did not use them for that purpose or mention PGD (Monk and Kathuria, 1977; Monk and Harper, 1978, 1979).

An immunological approach to mouse embryo sexing was reported by Krco and Goldberg (1976), who showed that exposure of embryos to an antiserum to the male histocompatibility-Y antigen resulted in lysis of male preimplantation embryos, leaving only female embryos. However, like Epstein et al. (1978), the authors did not suggest a clinical or commercial application for their method. This immunological approach was later applied to bovine embryos with a reported accuracy of 80–93% (cited in Beardsley, 1983; Shapley, 1983). Indeed, a patent for this method was filed in 1983 in America, providing an early example of the commercial use of sexing (Hare and Betteridge, 1983; Shapley, 1983).

These animal studies in the 1970s and early 1980s showed that PGD could be achieved in the absence of PCR, or even single cell biopsy and testing, although the accuracy and feasibility of these approaches was not tested on a large scale. However, only Modlinski and McLaren (1980) referred to its possible use in humans for either sexing or diagnosing other genetic characteristics or diseases, a theme also picked up in a news item in *Nature* entitled ‘Cattle now, people next?’ (Beardsley, 1983), in which Edwards was cited as finding the results of cattle sexing experiments ‘extremely interesting’. Otherwise, the possible human application of PGD was ignored and was not seriously engaged with again until 1986.

#### Pursuing PGD in human embryos

Whilst it is true that human embryos were hard to come by in the 1960s and 1970s (Table 2), Edwards and Steptoe made rapid progress in their culture of IVF embryos (Edwards and Steptoe, 1974; Edwards et al., 1970; Steptoe et al., 1971). So encouraging were the outcomes obtained by Edwards and Steptoe that they approached the MRC in 1970 for long-term support and formally submitted a grant application in February 1971 (Johnson et al., 2010). It is clear that Edwards and Steptoe believed that PGD was amenable to study and that adequate numbers of embryos could be generated for this purpose. Edwards (1971) suggested that the ‘next development’ in IVF studies ‘could well be the sexing of embryos before transfer and that this would be ‘an excellent approach to the control of sex-linked mutant genes in man’, and they included PGD in their grant proposal as one of two core objectives (NA1):

The basic research is helping our understanding of various aspects of human reproduction. Clinical application of the findings could lead to the alleviation of infertility in some cases, and might eventually provide the means for averting the birth of children with certain inherited disorders.

These aims were reiterated in a working party report of the British Association for the Advancement of Science (Jones and Bodmer, 1974, p. 31), of which Edwards was a member. In addition, a 1972 study by the British Medical Association reported on the potential to ‘diagnose certain fetal abnormalities in fertilized ova’ and that ‘this way might be preferable to termination at 16 weeks’ following amniocentesis (cited in Jones and Bodmer, 1974, pp. 120–121). Pembrey (1979), stimulated by a paper from Craft and Yovich (1979), addressed this question obliquely by proposing the use of embryo donation as a way of avoiding transmission of genetic disease. Moreover, sex selection by sperm sorting had also been tried unsuccessfully in humans, indicating interest in genetic selection (Schaffir, 1991). However, there was little enthusiasm for PGD itself. Indeed, the MRC declined to fund Edwards and Steptoe’s experiments (Johnson et al., 2010).

It was not until the mid-1980s that a clear motivation to achieve clinical PGD emerged. Thus, late in 1986, two meetings convened to discuss the prospects for achieving PGD were held: one at the CIBA Foundation (13 November, organized by David Whittingham and Richard Penketh) and one at the European Society for Human Reproduction and Embryology (ESHRE; 16 December in Strasbourg). These meetings were attended by several prominent UK players then pursuing human embryo research, many of whom engaged later in research to achieve PGD clinically (Table 3). The report on the CIBA Foundation meeting (Whittingham and Penketh, 1987) reviewed technologies for PGD and drew attention to both in-situ hybridization, which had recently been used to diagnose trisomy 21 by amniocentesis (Julien et al., 1986) and to PCR. The latter was discussed by Marcus Pembry in his 10 min talk entitled ‘Molecular diagnosis – the potential for gene amplification’ (Christie and Tansey, 2003, p. 53), and, in handwritten notes on the programme for this meeting, Anne McLaren (BL1) recorded that a paper on this topic had been published the same week in *Nature* (November 13–19; Saiki et al., 1986). Edwards is reported by Pembrey as jumping up and dancing around the room saying ‘75 cells, 75 cells, we are going to be able to do it!’ because he had earlier said ‘I don’t think it is on, we can’t give you geneticists enough cells’. (Christie and Tansey, 2003, p. 53). The meeting summary concluded that there was a clear clinical need for PGD and that research on human embryos was required to achieve it (Whittingham and Penketh, 1987).

The ESHRE meeting was stimulated, chaired and opened by Edwards (1987), who reviewed approaches to sex selection and PGD. At Edwards’ request, a potential diagnostic role for PCR was presented (Southern, 1987) based on the paper by Saiki et al. (1985; published 20 December) while Jones et al. (1987) presented their preliminary results using in-situ hybridization to sex human and mouse spermatozoa and whole preimplantation embryos. It is clear from the planning and proceedings of this meeting that Edwards had been galvanized into renewed activity regarding PGD (Brambati, 1987; Edwards and Hollands, 1988), including an exhortation to fight political pressures to ban the research that might make it possible (Anon, 1987).

Soon afterwards, the research pace increased. Non-invasive approaches to PGD were investigated as less technically and ethically challenging alternatives to embryo biopsy (Edwards, 1987), including measurement of pyruvate turnover to assess viability of whole human embryos and eggs (Hardy et al., 1989; Leese et al., 1986). PCR analysis on human and mouse oocytes, sperm cells and whole embryos indicated a practical route towards PGD (Coutelle et al., 1989; Li et al., 1988). Different experimental biopsy methods were investigated (Dokras et al., 1990; Summers et al., 1988; Verlinsky et al., 1990; Wilton and Trounson, 1989). A mouse model for PGD was developed, using single blastomere and trophoblast biopsies to sex embryos by assaying for the X-linked enzyme HPRT (Monk, 1988; Monk and Handyside, 1988; Monk et al., 1987, 1988). These experiments built on previous work by Monk and co-workers (Monk and Kathuria, 1977; Monk and Harper, 1978, 1979; Monk and McLaren, 1981; McMahon et al., 1981, 1983; McLaren and Monk, 1981; Harper and Monk, 1983), who, like Epstein (1969, 1970, 1978) had used X-linked enzyme assays to study dosage compensation and X inactivation in whole preimplantation mouse embryos, but, also like Epstein et al. (1978), had then made no reference to using this approach to type and transfer embryos for research, commercial or clinical purposes. In striking contrast, Monk and her colleagues clearly specified in their 1987 study the scope for applying PGD clinically and followed it by developing mouse models for adenosine deaminase deficiency and β-thalassaemia (Benson and Monk, 1988; Holding and Monk, 1989). Sexing of human IVF embryos by micro-enzyme assays (Braude et al., 1989), in-situ hybridization (Grifo et al., 1990; West et al., 1987) and Y-chromosome PCR (Handyside et al., 1989) were reported and pregnancy resulting from transfer of PCR-sexed human embryos followed (Handyside et al., 1990).

These human-oriented studies differed from the intervening work in animals in three key respects. First, many of them were published in journals with a wider inter-disciplinary audience, such as *Nature* and *The Lancet*, rather than in specialist agricultural journals such as *Theriogenology*. Second, whilst studies and discussions on human PGD did emphasize the potential for sexing to avoid X-linked genetic disease, they also expressed the hope that PGD could soon be extended to detect further genetic conditions. In contrast, earlier animal studies had focused heavily on preimplantation sexing for commercial and scientific reasons. Finally, these studies were clearly the result of a concerted effort to achieve PGD clinically.

### Conclusion

From 1986–1987 onwards, there was a quite sudden surge in interest and activity in clinical PGD. Whilst technological limitations undoubtedly posed difficulties from the late 1960s to the mid-1980s, after which possible solutions were beginning to emerge, the collective motivation to engage with PGD is not readily explicable in technological terms alone. How can the previous lack of interest in achieving clinical PGD be explained? And what had changed by 1986?

#### The motivation to achieve clinical PGD

Two broad factors seem to underlie this sudden change of motivation. First, changing attitudes to prenatal testing and IVF introduced a climate more favourably disposed to PGD. Second, political events in the UK that threatened scientists’ capacity to undertake research on human embryos provided a positive stimulus to actively pursue PGD.

#### Preventing genetic disease: PGD and prenatal testing

This study suggests that the apparent lack of research interest in developing PGD clinically in the 1970s was due, at least in part, to the parallel technological and clinical development of prenatal testing in obstetric medicine. Unlike PGD, prenatal testing did not require as-yet undeveloped single-cell testing techniques, but more significantly did not entail the unfamiliar technological or ethical challenges of IVF. Rather, it involved the development of surgical gynaecological techniques that were more familiar to, and so more readily accepted into, clinical practice.

Prenatal testing was initially performed by amniocentesis. Serr et al. (1955) first stained centrifuged amniotic fluid cells for sex chromatin, karyotyping being used later (Steele and Breg, 1966). The UK Abortion Act (1967) legalized termination of pregnancy on grounds of ‘substantial risk’ of serious physical or mental handicap, allowing for clinical intervention following amniocentesis. Indeed, the potential to diagnose and terminate affected pregnancies played an important role in the passage of the Abortion Act, many Parliamentarians emphasizing the ‘suffering caused to both children and parents by such disasters’ (Jenkins: Hansard, 1966). The widespread public and professional attention given to the thalidomide tragedy of the early 1960s ‘enabled abortion to be formulated as a public health issue’ (Lee, 1998, p. 79), at a time when doctors felt that they had ‘failed to keep their contract’ if a baby was born as ‘anything but normal’ (p. 167). Thus, at exactly the time that proof of principle of PGD was demonstrated, the options for prenatal testing were expanded.

**Figure 1:**
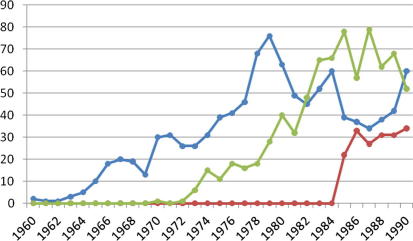
Plot of time course (by year) of PubMed search outcomes using, as search terms: amniocentesis (blue line); chorionic villus sampling (red line); ultrasound and prenatal diagnosis (green line). Plots give an indication of the novelty of the technology. (For interpretation of the references to colour in this figure legend, the reader is referred to the web version of this article.)

Prenatal testing was taken up somewhat slowly in clinical practice during the early 1970s (Figure 1; Milunsky, 1979, p. 19), although the number of diagnosable conditions increased steadily, and safety was demonstrated by large-scale clinical trials conducted in America, Canada and by the UK’s MRC (Milunsky, 1992, pp. 45–47). In addition, the experimental and clinical use of ultrasound in gynaecological and obstetric practice rose steeply from the early 1970s (Figure 1; White, 1982), making prenatal testing safer still. In the early 1980s, chorionic villus sampling (CVS) began to enter clinical practice (Christie and Tansey, 2003, p. 46; Milunsky, 1992, p. 124), offering a first-trimester alternative to later, and thus more physically and emotionally traumatic, second-trimester amniocentesis followed by abortion.

Despite the fact that prenatal testing was relatively new to clinical practice, it was viewed more favourably than PGD in the 1970s. For example, in their referees’ reports on Edwards and Steptoe’s 1971 grant application to the MRC, John Evans and Roger Short described PGD as ‘fanciful’ and ‘complicated and unnecessary’ and deemed amniocentesis followed by abortion to be a ‘far simpler’ approach (NA2). Indeed, the MRC, and much of the medico-scientific community, had reservations about IVF in general and felt that research on, or transfer of, IVF embryos would be unethical until they were provided with ‘satisfactory evidence that there would be no increased risk of abnormal offspring’ (NA3). It was not until the birth of a healthy IVF baby in 1978 that such attitudes towards IVF began to soften (Johnson et al., 2010). The MRC declared the need for an ‘urgent’ review of policy on funding IVF research in 1978–1979 (NA4) and identified the investigation of ‘inherited disease’ as one of the principal aims of embryo research, encouraging the pursuit of ‘a screening device for determining the chromosome constitution of an embryo’ (NA5). Moreover, key medico-scientific bodies suggested a range of approaches to achieving PGD, including ‘the removal of cells from the very early embryo’ (Royal Society, 1983) and ‘embryo division’ (RCOG, 1983). These bodies submitted their views to the Government-sponsored Committee of Inquiry into Human Fertilisation and Embryology (1982–1984; Warnock, 1984). Thus, from 1978 onwards, members of a handful of key organizations expressed an interest in pursuing embryo research to overcome genetic disease, both by PGD and by other approaches. However, as has been seen, it was not until 1986–1987 that this interest was reflected in scientific meetings and active research.

Indeed, negative attitudes towards PGD persisted into the early 1980s. For example, only two paragraphs among the 79 pages of the Warnock Report (Warnock, 1984) were devoted to PGD. These paragraphs were drafted by Anne McLaren (Warnock, unpublished interview), who felt ‘dubious’ about the possibility of ‘wide scale’ social sexing, and judged the detection of abnormalities by embryo biopsy as ‘unlikely’ to become feasible ‘for some considerable time’. Although described as a powerful advocate for embryo research on the Warnock committee (secretary Jenny Croft, unpublished interview), McLaren (1985) went on to submit a pessimistic review article on PGD in July 1984 and published in the January/February 1985 edition of *Prenatal Diagnosis*. She concluded that PGD could ‘only be considered as a last resort’ as it would further decrease the already very low success rate of IVF, and that CVS ‘would seem to offer better prospects’. Although she discussed various approaches to PGD in humans and animal models, McLaren deemed each to be fraught with difficulties: trophectoderm biopsy was unlikely to succeed ‘within present ethical constraints’; second polar body biopsy was ‘exacting’ and provided only ‘limited’ information; nuclear transplantation to provide biopsy material had been largely ‘unsuccessful’ in mouse models; and biopsy during cleavage would require ‘much research’ to ensure that normal development was not impeded.

Curiously, her perspective changed dramatically within only a few months. Writing with WH Evans – one of Edwards’ and Steptoe’s referees and thus another erstwhile critic of PGD (NA2) – McLaren declared in *Nature* on 14 March 1985 that the ability ‘to circumvent the production and transfer of severely genetically abnormal embryos’ was ‘almost within the grasp of medical science’ and the ‘means to undertake [it] just around the corner’ and ‘almost to hand’ (Evans and McLaren, 1985). McLaren’s enthusiasm for PGD was reiterated at a CIBA Foundation meeting on human embryo research that she had organized in November 1985 (some key participants listed in Table 3). This meeting focused mainly on embryo research for infertility treatment and contraceptive development and on the ethico-legal aspects thereof (Bock and O’Connor, 1986). PGD featured only briefly in a chapter on the diagnosis and management of genetic disease and was described as a technically difficult and unnecessary alternative to CVS (Bock and O’Connor, 1986, p. 97). However, in the discussion that followed (Bock and O’Connor, 1986, pp. 100–104), Edwards and McLaren were both more positive about PGD, while Modell deemed that it would be a ‘great benefit’ to high-risk couples faced with the alternative of ‘stressful’ terminations. A later chapter (Bock and O’Connor, 1986, p. 112) stressed the ‘real demand for diagnosis … during the preimplantation stages’, and in its discussion Braude (Bock and O’Connor, 1986, p. 115) was optimistic about future prospects for PGD.

The pleas from Modell and others suggest that the climate of medical opinion, propelled by patient pressure, was shifting away from prenatal diagnosis, with its attendant suffering, and was becoming more sympathetic towards PGD (Christie and Tansey, 2003; Rapp, 1999; Rothman, 1988, pp. 46–48). But was this transition driven simply by a greater familiarity with, and less antipathy towards, IVF? The final section in this paper suggests that political debates in 1984 and 1985 provided a strong positive stimulus for the proliferation of meetings and laboratory studies on clinical PGD that ensued from 1986 onwards.

#### Gaining political credibility: PGD and the UK embryo research debate

In the Parliamentary debates immediately following the publication of the Warnock Report (19 July; Warnock, 1984), the majority of Lords (31 October 1984) and MPs (23 November 1984) who spoke were firmly against human embryo research (see Table 4 for key political dates). The Marquess of Reading framed the Lords debate in terms of moral arguments, defining the ‘moral validity’ of embryo research as dependent on the ‘nature of the human embryo itself’ (Hansard, 1984a) and anti-research speakers rejected the Warnock Report as ‘defective’ precisely because it failed to ‘deal with the basic moral and ethical principle’ (Lord Rawlinson: Hansard, 1984a). Most speakers drew strongly on anti-abortion rhetoric, arguing that embryo research, like abortion, was ‘murder in a moral sense’ (Earl of Halsbury: Hansard, 1984a). The Commons debate was framed similarly (Abse: Hansard, 1984b). The Press picked up on this parliamentary hostility (LSE1).

Alarmed by the anti-research rhetoric, scientists responded. At the MRC, Joan Box sent a hastily drafted memo (NA6) to Malcolm Godfrey (Second Secretary, 1983–1988):

1. Dawes’ Advisory Group [NA7] have prepared a draft response [to the Warnock Report] indicating why research is necessary and suggesting joint action with RCOG to establish voluntary interim licensing arrangements – to be considered by Council on 29th November …

3. After the Lords debate Professor Callum Macnaughton (President of RCOG) concerned about the strength of feeling expressed in debate, suggested to Dr Box that he might approach you informally about a joint initiative …

4. Several other scientists including Dr Whittingham, Dr Lincoln, Dr Johnson and Dr Braude have expressed concern that urgent action on the public relations front is required – while public opinion is still being formed – to modify the biased picture emerging from the media and the Lords …

6. Norman Fowler [Secretary of State for Social Services, 1981–1987], worried about the pressure for a moratorium on research, is thought to be informing ministerial colleagues that he will approach you and the RCOG about the possibility of voluntary self-regulation being instituted urgently.

The memo continued by listing questions that had arisen, including:

1. Should MRC public relations be high profile or low profile?

2. Can scientists in the field proposing immediate action such as contacting local MPs, writing to ‘The Times’, speaking to the Press, etc meanwhile be reassured that you are happy that they do this in an individual capacity …?

The reply on 7 November 1984 (NA8) indicates that the memo had been discussed with Sir James Gowans (Secretary of the MRC) and confirmed that he was taking forward discussions on what would later become the Voluntary Licensing Authority (BL3), advised a low profile for the MRC and said that ‘Sir James would not wish to take any initiative about encouraging people to write to “The Times”, etc.’

Notwithstanding this last point, those who would be most adversely affected by a ban on research were already active. For example, earlier in 1984, Peter Braude (clinician), Martin Johnson and Hester Pratt (scientists) at the University of Cambridge had embarked on a 5-year programme of research on human and animal embryos funded by the UK Medical Research Council (MRC Annual Report for 1983/4), much of which risked becoming prohibited. During November and December 1984, they initiated a pro-embryo research campaign, contacting MPs (listed in LSE2) and contributing letters and articles in the media (LSE3).

On 5 December of that year, Ulster Unionist MP Enoch Powell introduced a Private Members’ Bill to ban all production and use of human embryos, other than to help an infertile woman to become pregnant. This galvanized further campaigning, such that Braude and his colleagues expanded their media activities (LSE4), contacted Government ministers and the cabinet office (LSE5) and organized an ‘all party discussion on embryo research in the House of Commons’ (15 January, LSE6). They joined forces with Joanna Chambers, the General Secretary of the Birth Control Campaign, a lobbying group with well-established political contacts (LSE7), and the Women’s Reproductive Rights Centre (LSE8). Despite their efforts and a letter from Gowans to *The Times* on the eve of the debate (LSE9), the Bill received a large Commons majority of 238:66 at its second reading on 15 February 1985 (Hansard, 1985). Shortly thereafter, Braude and Johnson (LSE10) wrote to Macnaughton and Gowans (copied to McLaren and David Whittingham, both heads of MRC Units), asking for more direct administrative support for the campaigning. The MRC, which in January 1985 had issued a revised policy statement on Research Related to Human Fertilisation and Embryology (LSE11, 1985), responded by assigning Dr Keith Gibson to help with the lobbying (LSE12). The group of scientists actively lobbying Parliament by now included Macnaughton, McLaren, Whittingham and Robert Winston (LSE13).

Mulkay (1997, p. 63) suggests that ‘in these early debates, reference to control of genetic disease had been a minor feature’ and that only ‘during later pro-research speeches’ did it become ‘the central topic’. However, a close examination of the February Commons debate on the Powell Bill (Hansard, 1985) reveals that all but one of 11 pro-research speakers referred to control of genetic disease, both by PGD and other approaches, while 10 of the 13 anti-research MPs responded to such claims. This was the result of the briefings that emphasized the potential clinical benefits of PGD by ‘embryo biopsy’ provided to pro-research Parliamentarians, including Leo Abse, David Crouch, Frank Dobson, Willie Hamilton, Jo Richardson, Peter Thurnham and Daffyd Wigley (CC1; Hansard, 1985).

However, the issue of PGD proved divisive amongst anti-research speakers in the Commons debate on the Powell Bill. Some disagreed with PGD in principle and argued that, in caring for handicapped children, ‘individuals and the community’ were made ‘that much greater’ (Beith: Hansard, 1985). However, most did not oppose the principle of handicap prevention, just the use and destruction of embryos to achieve it, equating the embryo with an ‘unborn child’ and so claiming that the potential benefits to ‘other human beings’ (Winterton: Hansard, 1985) could never be justified. Others were concerned that the ‘rectification of genetic defects’ taken to ‘its logical conclusion’, selection for characteristics such as IQ and skin colour would follow, such that prospective parents could ‘book’ a ‘tall, dark, handsome’ baby or even ‘a Frankenstein monster’ (Campbell-Savours: Hansard, 1985). Still others doubted that embryo research could in fact lead to clinical PGD (Braine: Hansard, 1985) and suggested that research on human gametes and animal embryos would be more fruitful and more ethical (Tracey: Hansard, 1985). Six anti-research speakers supported this stance by citing the highly-publicized views of pro-life French geneticist Jerome Lejeune (21 February; Walgate, 1985). Lejeune had flown in from Paris to address the MPs (Braine: Hansard, 1985) and had claimed that PGD could not work, a claim later challenged in a letter to *The Times* by eight leading geneticists (Bobrow et al., 1985).

Powell himself took a somewhat different stance to many anti-research speakers, treading a fine line between endorsing IVF and PGD whilst opposing destructive research. Although he was one of only three anti-research speakers in the February Commons debate who did not refer directly to the control of genetic disease, his subsequent private and public communications emphasized his strong desire that the Bill should allow ‘embryo biopsy’ (CC1) to diagnose and exclude abnormal embryos. If, according to Powell, there was ‘a clinical ethical duty not to insert an unsatisfactory embryo’, then ‘there must be a right (or duty) within the Bill’ to allow tests ‘to detect unsatisfactory embryos’ (CC2). Powell asserted that such tests would only be precluded ‘if they required the creation and use of IVF embryos solely for the purposes of research’, rather than transfer (CC3). As such, his view was in diametric opposition to that of the Warnock Report (1984), which stated ‘no embryo which has been used for research should be transferred to a woman’.

In March 1985, Powell sought advice on the impact of his Bill on PGD from Roy Cunningham, Assistant Secretary at the Department of Health, who later was to lead the team of policy officials involved in drafting the HFE Bill (Anon, 2008; Cunningham, 1991). Cunningham confirmed: ‘the [Powell] Bill as it stands would allow such procedures [biopsy]’ (CC4). Later that month, however, Cunningham warned Powell that the achievement of clinical PGD would first ‘depend on further research’, which might fall outside the limits of the Bill (CC2). By then, however, Powell had already announced to the Parliamentary Standing Committee that he had been ‘assured in writing’ that ‘embryonic biopsy could continue’ under his Bill (CC1).

Powell’s stance on PGD fractured the anti-research campaigners. The Order of Christian Unity warned that the Bill would ‘not achieve effective protection for the human embryo’ (CC5), while David Poole, chairman of the Association of Lawyers for the Defence of the Unborn, could ‘no longer support’ or ‘even conceal [his] opposition’ to the Bill, as it would allow embryos with ‘genetic imperfection’ to be ‘discarded and destroyed’ (CC6). In addition, although Powell was the only speaker in the Standing Committee debate in March to stress that PGD would be allowed under his Bill, some of his closest colleagues subsequently echoed his stance. For example, Bernard Braine, in a letter to geneticist Martin Bobrow, confirmed that ‘there is nothing in the … Bill to stop embryonic biopsy’, but somewhat incongruously stated in the same paragraph that ‘there is no way’ that the ‘destruction of the [diagnosed] embryo could be described as a ‘cure’’, perhaps indicating his own ambivalence towards PGD (CC7).

Powell’s stance, and its impact on his allies, alerted pro-research Parliamentarians and lobbyists to a potential weakness in the anti-research case. They accused him of being ‘totally disingenuous’ and maintained that destructive embryo research was ‘essential’ before PGD ‘could ethically be used in a clinical context’ (Hamilton, CC1). Indeed, MP Peter Thurnham wrote at least three letters over the next 2 years to Powell and Phyllis Bowman of the Society for the Protection of the Unborn (SPUC), challenging Powell’s claim to the Standing Committee that the Bill would ‘in no way interfere with the practice of IVF procedures’ or their ‘improvement’ (CC8). The potential benefits of PGD also appeared to play well with the public and may have further stimulated the pro-research lobby to doggedly pursue the issue. Thus, in April 1985, a Marplan poll commissioned by Middlesex Polytechnic reported that ‘nearly half of the opponents [of embryo research] changed their mind when asked if they would favour research if it would help eliminate genetic diseases’, shifting the totals in support of such research from 32 to 51% (Clarke, 1985). Articles appeared in the Press by, or quoting, eminent geneticists describing the potential of PGD (LSE14).

The response of the anti-research lobby, including Powell, was to demand that the scientists ‘provide detailed information of research projects (particularly those relating to genetic diseases)’ which would be precluded by the Bill, but, according to the All-Party Parliamentary Pro-Life Group in a letter to *The Times*, they received only ‘vague conjectures’ (CC9). Indeed, in a *Nature* editorial in March 1985, the scientists were advised that they could ‘help themselves and even help defeat the bill’ by publishing their embryo research proposals (Anon, 1985). At a rally of the SPUC 4 months later, Powell demanded that ‘the government and the professions must come clean with the public’ and painted the parents of genetically handicapped children as gullible, having displayed ‘their private griefs in lieu of logical argument’ (CC10). Might it have been these direct challenges to scientists that provided the motivation for them to engage intellectually and practically with PGD?

Notwithstanding these challenges, by November 1985, when the pro-research lobby had consolidated formally into the campaign group Progress, it identified from the outset the ‘prevention of … congenital handicap’ as one of three main objectives of embryo research (LSE7) and received strong support from numerous disability charities, including MIND, SENSE and MENCAP (LSE11; Rix et al., 1985). Indeed, Progress increasingly emphasized PGD in their lobbying, such that by January 1988 in a brief distributed to all MPs, Progress devoted only five lines to IVF treatment but 28 lines to ‘preventing handicap’ (LSE13).

This political focus on PGD was reinforced by the concurrent discussions on termination of pregnancy. In the 1980s, abortion was debated in parallel with embryo research, as Parliament considered whether to reduce the time limit of legal terminations from 28 weeks of pregnancy to 18, 20, 22, 24 or 26 weeks. Parliamentarians on both sides centred their case on late abortions performed on grounds of fetal abnormality. Anti-abortion speakers suggested that ultrasound and CVS could allow diagnosis as early as 9–10 weeks (Lord Bishop of Birmingham: Hansard, 1987), obviating the need for late abortions following amniocentesis. In contrast, those against reducing the legal time limit argued that CVS was ‘not universally available’ and that its safety had not been confirmed (Newton: Hansard, 1988a). They maintained that, ‘although few in number’, those late abortions following amniocentesis were ‘important because a severely handicapped child can cause immense personal and domestic problems’ (Lord Butterworth: Hansard, 1988b). Crucially, several pro-research Parliamentarians highlighted the potential for PGD to provide an alternative, emphasizing the ‘illogicality of some Honourable Members’ who supported reducing the legal time limits, ‘but at the same time’ opposed the research that would ‘prevent the necessity for late abortions’ (Rhodes James: Hansard, 1988c). In doing so, the potential clinical benefits of PGD were re-iterated, gaining further political credibility.

## Discussion

This article has traced the history of the development of PGD from initial animal studies in the late 1960s to its clinical application in 1990. It has found that both its development and application in humans is partially explicable through the technological difficulties posed by species differences, limited human embryo availability and the requirement for sensitive tests. However, these difficulties alone do not adequately explain the patterns of activity observed. Attempts were made to overcome the technical hurdles in farm animals in the 1970s and early 1980s, but not in humans, suggesting a lack of motivation to achieve PGD clinically. The evidence suggests that through the 1970s this lack of interest may have been due to the parallel development and application of clinical prenatal diagnostic technologies. These were technically and conceptually more familiar to clinicians than IVF and embryo research, both of which generated strong medico-scientific reservations (Johnson et al., 2010). However, by the mid-1980s, IVF babies were being born in increasing numbers (HFEA, 2010) and in several countries (Cohen et al., 2005), and IVF itself had gained greater acceptance both publicly and professionally – as shown in the MRC’s 1982 decision to fund research into IVF (Johnson et al., 2010). Indeed Powell himself, like many of his politically aligned colleagues, supported IVF as a treatment for the infertile.

However, changing attitudes to IVF do not in themselves explain why interest in pursuing PGD took off only from 1986. The motivation to work on this technique seems unlikely to have come from the work on large farm animals because, whilst almost all of the later experimental studies cited Gardner and Edwards’ 1968 paper, almost none cited the intervening farm animal work despite obvious conceptual and methodological similarities. Likewise, the advent of the PCR technique seems an unlikely, if much touted, stimulus. Thus, although the first paper applying PCR was published in December 1985 (Saiki et al., 1985), it reported use of template DNA from the equivalent of 600 diploid genomes. A paper in November 1986 lowered this figure to 150 nuclei (Saiki et al., 1986), but it was another year before the detailed method was published (Mullis and Faloona, 1987) and 2 years before single cell PCR was reported (Li et al., 1988). Perhaps not surprisingly, therefore, this methodology seemed to percolate only gradually into the collective consciousness of those discussing PGD during the 1986 CIBA and ESHRE meetings, and even then as one of several possibilities under consideration (Edwards, 1987). So, whilst it is undoubtedly the case that technological limitations posed difficulties from the late 1960s to the mid-1980s, and that possible technical solutions began to emerge in the late 1980s, the collective interest in PGD was already evident before this happened. This change in interest was most vividly demonstrated, and pinned in time to early 1985, in the publications of McLaren, when she transformed from a PGD sceptic to PGD enthusiast. She did so at exactly the time that the hostile Parliamentary responses to the Warnock report and the Powell Bill were threatening medical research. Powell did not want to stop IVF as a treatment for the infertile: it was the use of embryos in research to which he objected as being both unethical and unnecessary. This objection to research provided the focus around which a coalition of supporters rapidly formed to generate a powerful Parliamentary opposition, who, like Powell, appeared impervious to the fact that IVF was based on human embryo research.

McLaren’s response was important, because she was influentially placed. She had been a member of the Warnock Committee and was a respected player in the MRC and Royal College of Obstetricians and Gynaecologists (RCOG). She was also an articulate public defender of embryo research and PGD, for example by publishing in *Nature*, a science journal calculated to command a much wider audience than the esoteric journals used by those working on large farm animals. She was also prepared to talk to the opposition, having lectured on the status of the human embryo at the September 1984 AGM of LIFE, an anti-abortion organization (Bl3). However, she was not alone. The archival evidence shows the depth of concern generated in the scientific community by the Parliamentary reactions to human embryo research. Significantly, this concern was not limited to the small group of scientists directly concerned with embryo research, but was also evident in the higher echelons of the MRC and the RCOG. From this concern, a pro-research lobby alliance was hastily assembled, initially composed of scientists, doctors and MPs, but soon attracting medical charities, patients and pro-choice women’s groups.

The Parliamentary response threatened the development of IVF as a safe medical technology, based on the moral case made by opponents of human embryo research. PGD emerged as a strategic weapon with the potential strength to divide and defeat this opposition. The evidence illustrates the fracture lines that PGD opened within the opposition, alienating Powell from some of his key supporters. Whilst this paper agrees with Mulkay that PGD acquired ever greater political importance as the debate wore on, such that by 1989, 75% of pro-research Lords speakers made significant reference to the regulation of genetic disease, compared with only 50% to infertility treatment (Mulkay, 1997, p. 187), it differs from him in the finding that this political focus was evident from the outset and in the direct challenges that each side levelled at their opponents. Crucially, the challenge by Powell to prove that research was required for PGD to be developed preceded the pivotal clinically oriented PGD interest of 1986–1987, and, it is suggested here, stimulated it. Indeed, as has been seen, key members of the pro-research lobby subsequently participated in the ESHRE and CIBA meetings on PGD and many were involved in laboratory investigations on PGD in the late 1980s (Table 3). Such key players were then, as a direct result of their lobbying experience and contacts, well placed to communicate their research progress to both public and parliamentarians between 1985 and 1990, reaffirming the political focus on PGD. Several also recognized the need to ‘get together to influence the European Parliament’ in order to prevent a pan-European wave of human embryo research prohibition, stressing that the ‘overwhelming clinical need to do something to help families at high genetic risk’ using PGD should be emphasized politically throughout Europe (Penketh cited in Anon, 1987). Unsurprisingly, however, the argument that PGD offered moral advantages over later abortion had less force in Catholic Europe than in the UK (Anon, 1987), which was therefore strongly placed both ethically and practically to develop PGD through to clinical application.

In conclusion, this paper argues that, whilst the concept of PGD did indeed shape the course and outcome of Parliamentary debates leading to the HFE Act of 1990, those debates themselves exerted a powerful stimulus to the development of PGD as a novel technology. The scientific community perhaps should acknowledge a debt to Powell for his positive influence on the emergence of this new medical technology.

## Figures and Tables

**Table 1:** Comparison of the first experimental and clinical demonstrations of PGD.

*Comparison*	*First experimental demonstration* (*Gardner and Edwards, 1968*)	*First clinical application* (*Handyside et al., 1990*)
Species	Rabbit (2.5-mm diameter embryo)	Human (150 mm diameter embryo)
Fertilization	*In vivo* and recovery from uterus	IVF
Embryo biopsy	Trophoblast biopsy (200–300 cells)	Single blastomere biopsy (1 cell)
Diagnosis	Sex chromatin visualization	Y chromosome-specific PCR
Embryo age	Biopsied 5.75 days after fertilization	Biopsied 3 days after fertilization
Embryos used	121 embryos from 15 rabbits	63 embryos from 112 eggs, 10 cycles with five couples
Sexing^a^	119 (98%) embryos biopsied, 104 (86%) embryos sexed	50 (79%) embryos biopsied, 46 (73%) embryos sexed
Embryos transferred^a^	40 (33%) embryos implanted, 24 fetuses at term (20%)	17 embryos transferred (27%), two twin pregnancies (6.3%)
Accuracy	18/18 offspring correctly sexed (confirmed at full term)	4/4 fetuses correctly sexed (confirmed by CVS at 10 weeks)

CVS = chorionic villus sampling.

a Percentage of fertilized embryos used.

**Table 2:** Time line of sources of human eggs and embryos.

*Author(s) year*	*Method*	*Patients*	*Eggs/embryos*
Rock and Menkin (1944)	Laparotomy + IVF^a^	3	18 eggs inseminated; 4/18 eggs ⩾2 cells^a^
Rock and Hertig (1948)	Hysterectomy	122	26 embryos
Hertig et al. (1959)	Hysterectomy	210	34 embryos
Edwards (1965)	Ovarian biopsy	16	250 unfertilized eggs
Edwards et al. (1969)	Ovarian biopsy + IVF	NA	56 eggs inseminated; 7/56 eggs ⩾2 pronuclei
Edwards et al. (1970)	Laparoscopy + IVF	49	212 eggs inseminated; 38/212 eggs ⩾2 cells
Clewe et al. (1971)	Hysterectomy	9	9 eggs collected; 1/9 eggs 2 pronuclei
Croxatto et al. (1972)	Uterine lavage	42	5 unfertilized eggs and 3 embryos
Lopata et al. (1974)	Laparoscopy or laparotomy	70	217 unfertilized eggs
Buster et al. (1983)	Donor uterine lavage + transfer to recipient	14	5 eggs and 5 embryos; 2 pregnancies
Buster et al. (1985)	Donor uterine lavage + transfer to recipient	5	25 embryos; 3 pregnancies
Formigli et al. (1987)	Donor uterine lavage + transfer to recipient	42	23 embryos; 8 pregnancies
Formigli et al. (1990)	Donor uterine lavage + transfer to recipient	127	48 embryos; 18 pregnancies

NA = not applicable.

a IVF claimed but subsequently disputed.

**Table 3:** Key players involved in human embryo research and/or discussion and research on human preimplantation genetic diagnosis (PGD) who attended CIBA and/or European Society of Human Reproduction and Embryology (ESHRE) meetings in 1985–1986.

*Name (location)*	*CIBA meeting: embryo research (11 June 1985)*	*CIBA meeting: PGD (13 November 1986)*	*ESHRE meeting: PGD (16 December 1986)*
J Aitken (Edinburgh)	+	–	–
D Baird (Edinburgh)	+	–	–
P Braude (Cambridge)	+	–	–
R Edwards (Cambridge)	+	+	+
A Handyside (Carshalton)	–	+	–
M Johnson (Cambridge)	+	+	–
K Jones (Edinburgh)	–	–	+
H Leese (York)	–	–	+
M Macnaughton (London)	–	+	–
J Maddox (*Nature*)	+	–	–
A McLaren (London)	+	+	+
B Modell (London)	+	+	+
M Monk (London)	–	+	–
A Muggleton-Harris (Carshalton)	–	+	–
R Penketh (London)	–	–	+
M Pembry (London)	–	+	–
D Whittingham (Surrey)	–	–	+
B Williamson (London)	+	–	–
R Winston (London)	–	+	–

+ = attended meeting.

**Table 4:** Key political events in the embryo research debate, 1984–1985.

*Date*	*Summary of main events*
July 1984	Publication of the Warnock Report
31 October 1984	Lords debate on the Warnock Report
23 November 1984	Commons debate on the Warnock Report
27 November–29 December 1984	Contacts with MPs to encourage support for embryo research
5 December 1984	Unborn Children (Protection) Bill tabled by Enoch Powell
15 January 1985	Pro-research all-party discussion in the House of Commons
15 February 1985	Second Reading Commons debate on the Powell Bill
March 1985	Voluntary Licensing Authority formed by the MRC and RCOG
6–20 March 1985	Standing Committee D debates on the Powell Bill
3 May 1985	Report Stage debate on the Powell Bill
7 June 1985	Commons MPs present 13 petitions with 4065 signatures against the Powell Bill; Powell Bill fails to secure extra time
23 July 1985	Meeting of the pro-research Warnock Legislation Group
6 November 1985	CIBA Foundation meeting on embryo research
12 November 1985	Progress founded by pro-research lobby members
